# Assistive Grasping Based on Laser-point Detection with Application to Wheelchair-mounted Robotic Arms

**DOI:** 10.3390/s19020303

**Published:** 2019-01-14

**Authors:** Ming Zhong, Yanqiang Zhang, Xi Yang, Yufeng Yao, Junlong Guo, Yaping Wang, Yaxin Liu

**Affiliations:** 1 Industrial Research Institute of Robotics and Intelligent Equipment, Harbin Institute of Technology, Weihai 264209, China; zhongming@hit.edu.cn (M.Z.); 15732031132@163.com (Y.Z.); yangxi_hit@163.com (X.Y.); Yyf1023@163.com(Y.Y.); junlongg@hit.edu.cn (J.G.); 2 Department of Industrial Engineering, University of Houston, Houston, TX 77004, USA; ypwang@uh.edu

**Keywords:** wheelchair-mounted robotic arm, human-robot interaction, laser point, CNN

## Abstract

As the aging of the population becomes more severe, wheelchair-mounted robotic arms (WMRAs) are gaining an increased amount of attention. Laser pointer interactions are an attractive method enabling humans to unambiguously point out objects and pick them up. In addition, they bring about a greater sense of participation in the interaction process as an intuitive interaction mode. However, the issue of human–robot interactions remains to be properly tackled, and traditional laser point interactions still suffer from poor real-time performance and low accuracy amid dynamic backgrounds. In this study, combined with an advanced laser point detection method and an improved pose estimation algorithm, a laser pointer is used to facilitate the interactions between humans and a WMRA in an indoor environment. Assistive grasping using a laser selection consists of two key steps. In the first step, the images captured using an RGB-D camera are pre-processed, and then fed to a convolutional neural network (CNN) to determine the 2D coordinates of the laser point and objects within the image. Meanwhile, the centroid coordinates of the selected object are also obtained using the depth information. In this way, the object to be picked up and its location are determined. The experimental results show that the laser point can be detected with almost 100% accuracy in a complex environment. In the second step, a compound pose-estimation algorithm aiming at a sparse use of multi-view templates is applied, which consists of both coarse- and precise-matching of the target to the template objects, greatly improving the grasping performance. The proposed algorithms were implemented on a Kinova Jaco robotic arm, and the experimental results demonstrate their effectiveness. Compared with commonly accepted methods, the time consumption of the pose generation can be reduced from 5.36 to 4.43 s, and synchronously, the pose estimation error is significantly improved from 21.31% to 3.91%.

## 1. Introduction

With the wide application of robots and the large demand for intelligent robots to enhance the living quality of particular users [1,2], many methods have been applied to facilitate human–robot interactions, including gesture, face, laser point, mobile phone, and brain-machine interactions. Lee et al. [3] proposed a human–robot interaction method utilizing gesture recognition. Tanaka et al. [4] implemented a face recognition function that can robustly identify the user’s face and predict the face position; in addition, the assistive robotic arm will eventually be able to bring a cup to the user’s mouth based on the face recognition results. A stationary tabletop rehabilitation robot developed at the University of Delaware incorporates a laser pointer with which the user can select a few well-modeled objects, allowing the robot to focus its attention on the object [5]. Rouanet et al. [6] used mobile phones as a human–robot interaction method to guide a robotic arm to complete a grasping motion by circling objects on the mobile interface. Choi et al. [7] proposed techniques for controlling brain–machine interfaces using the higher human cognitive activity in a non-invasive manner, which can be used to rehabilitate or improve the cognitive performance of neurological or psychiatric patients with prefrontal dysfunctions.

Unlike the aforementioned methods, laser pointer interactions bring about a greater sense of participation in the interaction process as an intuitive interaction mode. Imtiaz et al. [8] used a remote specialist to control a laser pointer to improve the application of a teleconsultation. Kang et al. [9] developed a laser-pointer system for human–robot interactions, in which the user can draw trajectories and send commands. Karvelis et al. [10] instructed patients to follow a zig-zag pattern using a hand-held laser pointer to assess their sensorimotor function. Fukuda et al. [11] used a laser pointer to guide wheelchairs passing through various obstacles. Gualtieri et al. [12] used a four-layer deep convolutional neural network to grasp objects selected by a laser pointer.

In addition, researchers at Georgia Tech used a laser pointer to control a mobile platform and grasp large objects, and mounted a robotic arm, color camera, and depth camera on the platform [13,14]. In 2010, they upgraded the platform using a laser range finder to measure the 3D point cloud of different objects [15]. Although this system is delicate and can grasp an object as small as a vitamin tablet, the grasping success rate is only 58%.

For interaction with a laser point, a target matching method and background difference method are usually used for positioning. The target matching method uses the brightness [16], color [17], and shape [18] to detect an object, making it vulnerable to changes in illumination and distance. In addition, the method often misjudges the laser point because it is too small and becomes deformed. The background difference method utilizes the difference between the foreground and background frame images to detect regular changes [19,20], which requires a lengthy amount of time. In conclusion, there are still problems with laser point interactions, such as misjudgments and a poor real-time performance.

The deep learning method is popular in tracking, sensoring, and object classification, and a CNN has demonstrated a high performance in object detection and classification [21,22,23,24]. In this paper, a CNN is used to solve such problems as a misjudgment, poor real-time performance, and laser point and object detection issues in front of a dynamic background. The images captured using an RGB-D camera are first pre-processed to enhance the robustness, and then fed to a CNN to output the coordinates of the laser point and the selected objects. Moreover, the image output from the last layer of the CNN is visualized to ensure whether the object has been successfully selected. Ultimately, the object can be located within a 3D coordinate frame using the depth point cloud information once it has been correctly selected. In addition, assistive grasping experiments based on the laser-point detection method were carried out using a Kinova Jaco robotic arm. The rest of this paper is organized as follows: Section 2 introduces the proposed laser-point detection method. Section 3 describes the object grasping approach. Section 4 discusses the experimental verification results. Finally, some concluding remarks and areas of future work are presented in Section 5.

## 2. Laser point Detection

**Figure 1:**
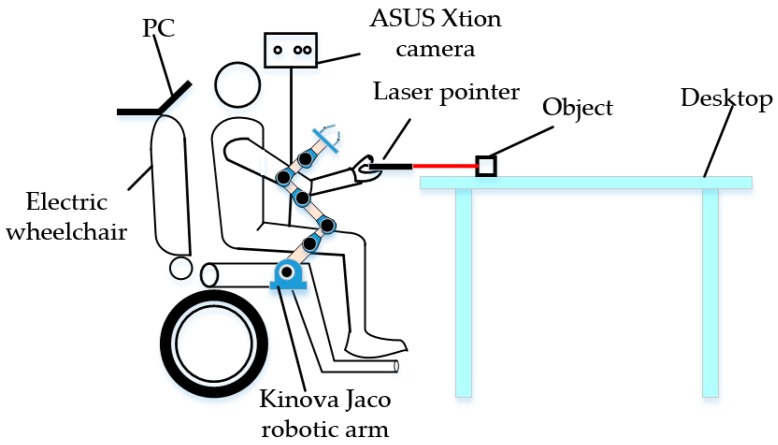
Diagram of experimental platform.

The human–robot interaction system described in this paper is composed of a PC, an electric wheelchair, an ASUS Xtion camera, a laser pointer, and a Kinova Jaco robotic arm, as illustrated in Figure 1. The human–robot interaction is fulfilled by first detecting the laser point, and then determining the object and grasping pose.

### 2.1. Image Pre-Processing

**Figure 2:**
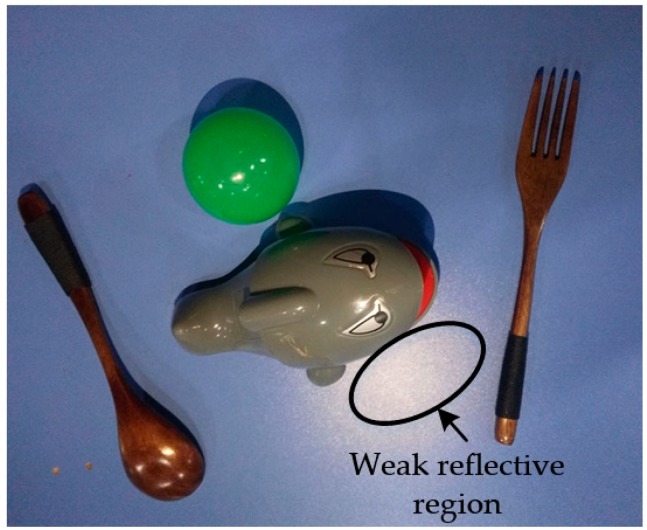
Weak reflective region.

**Figure 3:**
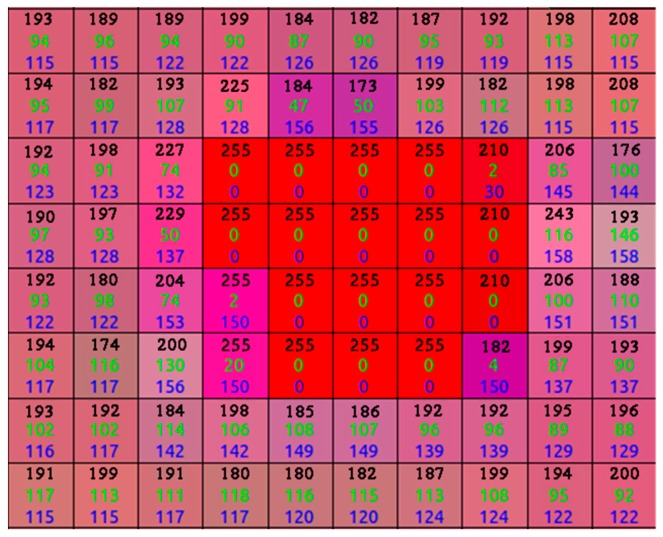
Laser point shown in HSV image using (255, 0, 0).

The images obtained using an AsusTek (ASUS, Taipei, Taiwan) Xtion camera with a pixel resolution of only 640 × 480 should be pre-processed prior to being fed to the CNN to increase the detection accuracy. A median filter is first used to eliminate the salt and pepper noise. Next, the images are converted from RGB into an HSV color space to weaken the influence of weak reflective regions (see Figure 2) at the pixel level. The pixels whose H, S, and V values are equal to those of the laser point (255, 0, 0) are considered a strong reflective region, and the pixels whose S values range from zero to 40 are deemed a weak reflective region. In the HSV color space, the S and V values of the laser-point pixels are both zero, as shown in Figure 3, whereas a value of 50 is added to the S values of the pixels in a weak reflective region that are greater than zero but less than 40. The modified HSV images are converted back into an RGB color space after the process described above. However, the laser-point detection algorithm still suffers from a strong reflective region, in which the S and V values are both zero. To solve this problem, a visualization module was added to a recently published CNN, as detailed in the following subsection.

### 2.2. Laser-Point Detection

A deep-learning-based algorithm can be divided into region-free and region-based methods. A Single Shot MultiBox Detector (SSD) and You Only Look Once (YOLO) are representative methods of the former, whereas a region-based convolutional neural network (R-CNN) and SPP-Net are representative of the latter [25]. A high detection accuracy is always accompanied with higher computational costs with regard to training and detection [26,27], and a region-free method is faster than a region-based method but at the cost of lower accuracy.

**Figure 4:**
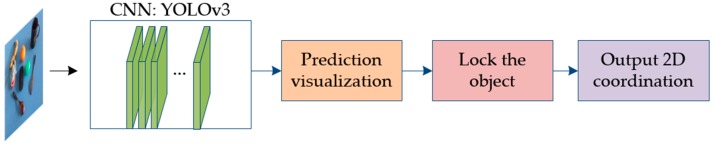
Detection flowchart of 2D coordinates of pointed object.

Both the objects and the laser point, which is made up of only dozens of pixels, need to be identified at the same time. Therefore, our experiment required both real-time and small-target object detection capabilities. Currently, among the above networks, only YOLOv3 and SSD are capable of achieving both simultaneously; YOLOv3 is as accurate as SSD but 3-times faster [28]. Owing to the steerable properties of a CNN [29], YOLOv3 was temporarily applied during the experiment. Eight kinds of objects were chosen for detection in an indoor environment: a banana, an orange, a ball, a toy, a mouse, a cup, a fork, and a spoon. A dataset containing 1000 photos of these objects and a laser point was established, and the images were obtained using an ASUS Xtion camera. The images were fed into the CNN, and the last layer of YOLOv3 (Figure 4) was visualized to help the user know whether the object had been successfully selected, the process of which is described in the following:

The CNN not only deals with image information of the objects and laser point, but also frames the detected objects. The sizes and shapes of the detected objects are dynamically changeable owing to variations in pose, deformations, and occlusions [30], which can be dampened through visualization.

**Figure 5:**
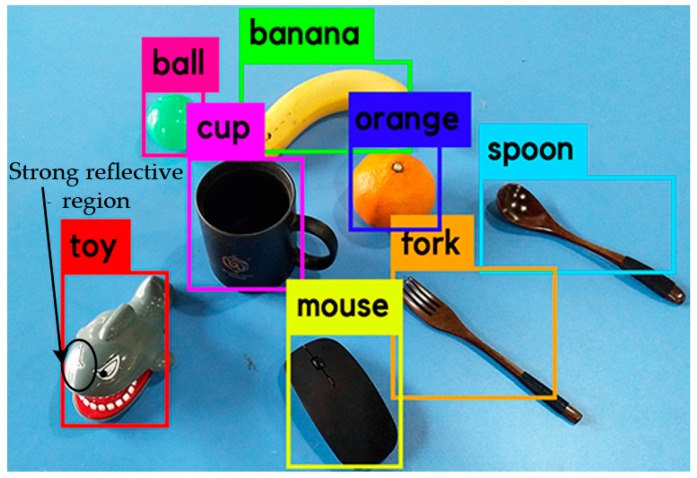
Diagram before object has been “locked”.

The frame of the laser point should be contained within the frame of the object being pointed at. If this relationship is satisfied, the object will be “locked,” and its frame color will simultaneously change. Next, the user can know whether the object being pointed at has been successfully selected. Moreover, if there is a strong reflection area on the object’s surface, the user can avoid guiding the laser point to this area (see Figure 5). Choosing the appropriate region by following a manual procedure does not seem to be the best approach for practical applications because the H, S, and V values of the pixels in a strong reflection area are the same as those of the laser point, and it may not be possible to solve this problem through image processing.

In conclusion, visualization can help ensure that the object has been correctly selected, and enhance the user’s involvement during the human–robot interaction. With the exception of in non-controlled environments with strong external illumination, the proposed laser pointing scheme performs well in indoor environments.

Beyond the detection operations described above, YOLOv3 also outputs the pixel coordinates of the object’s frame, which can be approximated as its 2D centroid coordinates. Combining the object’s 2D centroid coordinates with the depth information yields its 3D coordinates, which offers inputs to the grasping assignments of the Kinova Jaco arm.

## 3. Object Grasping

Object grasping can be fulfilled by first determining the object using its 3D coordinates and then the grasping pose, as detailed in the following two subsections.

### 3.1. Object Determination

**Figure 6:**
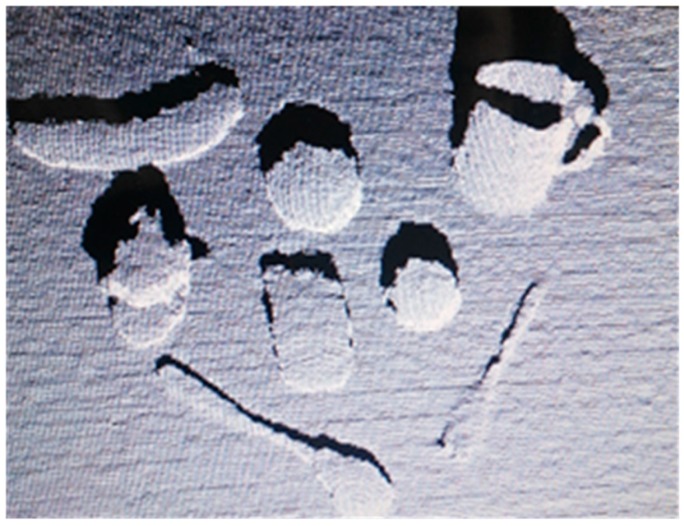
Point cloud image of grasped objects.

The point cloud data are acquired using an RGB-D camera, as shown in Figure 6. The X-, Y-, and Z-axis ranges of the camera are first restrained using a pass-through filter to remove unnecessary point cloud data, and to contain the target object. 

**Figure 7:**
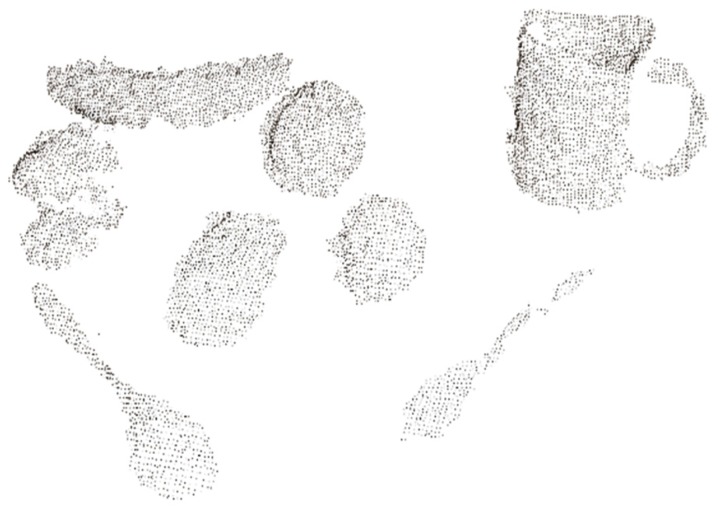
Object image after Euclidean cluster extraction has been applied.

A statistical filter and a voxel filter are then used to filter the outliers and further reduce the amount of point cloud data. After the process described above has been applied, the point cloud data are further processed using plane segmentation and target extraction to separate the objects, as shown in Figure 7. Because all target objects are on a desktop, the plane segmentation (using random sample consensus (RANSAC)) and target extraction (Euclidean cluster extraction) are selected. The RANSAC is first used to model the input point cloud data to eliminate inliers, and Euclidean cluster extraction is then used to separate the point cloud data of the objects.

The 3D centroid coordinates of the separated objects are first calculated, and then combined with the “locked” object’s 2D coordinates to finish the object determination task. The specific calculation formula is as follows:

(1) $$\left\{ \begin{array}{l} {\left(X_{0}-\frac{X_{i}}{Z_{i}}\right)}^{2}+{\left(Y_{0}-\frac{Y_{i}}{Z_{i}}\right)}^{2}<T\;\mathrm{Yes}\;\mathrm{It}\;\mathrm{is}\;\mathrm{the}\;\mathrm{object}\;\mathrm{to}\;\mathrm{be}\;\mathrm{grasped}\\ {\left(X_{0}-\frac{X_{i}}{Z_{i}}\right)}^{2}+{\left(Y_{0}-\frac{Y_{i}}{Z_{i}}\right)}^{2}>T\;\mathrm{No}\;\mathrm{It}\;\mathrm{is}\;\mathrm{not}\;\mathrm{the}\;\mathrm{object}\;\mathrm{to}\;\mathrm{be}\;\mathrm{grasped} \end{array}\right.$$

where $X_{0}$ = “locked” object’s 2D X coordinate component;

$X_{i}$ = objects’ 3D X coordinate component;
$Y_{0}$ = “locked” object’s 2D Y coordinate component;
$Y_{i}$ = objects’ 3D Y coordinate component;
$Z_{i}$ = objects’ 3D Z coordinate component; and
*T* = the threshold.

### 3.2. Grasping Pose Determination

There are many pose estimation algorithms based on 3D point cloud images, including a template-selected method using global features, a local feature matching method, and an iterative closest point (ICP) algorithm. The template-selected method can determine a template object from a template library, which is similar to the selected object, for example, using a point feature histogram (VFH) and a clustered viewpoint feature histogram (CVFH) [31,32]. These methods can be used to recognize objects and estimate their pose, and the time consumption is acceptable. However, the pose estimation accuracy for a sparse template library is low. The local feature matching method, however, easily incurs an incorrect match. Although the ICP algorithm is the most accurate at determining an object’s pose, its time consumption is high.

To deal with a sparse multi-view template library, this study uses coarse- and precise-matching of the target to the template objects. The coarse-matching method is first used to select from the library the template object that is the most similar to the object being pointed at. Then, the precise-matching method is used to calculate the precise pose of the object.

### 3.3. Coarse-Matching of Target to Template Objects

**Figure 8:**
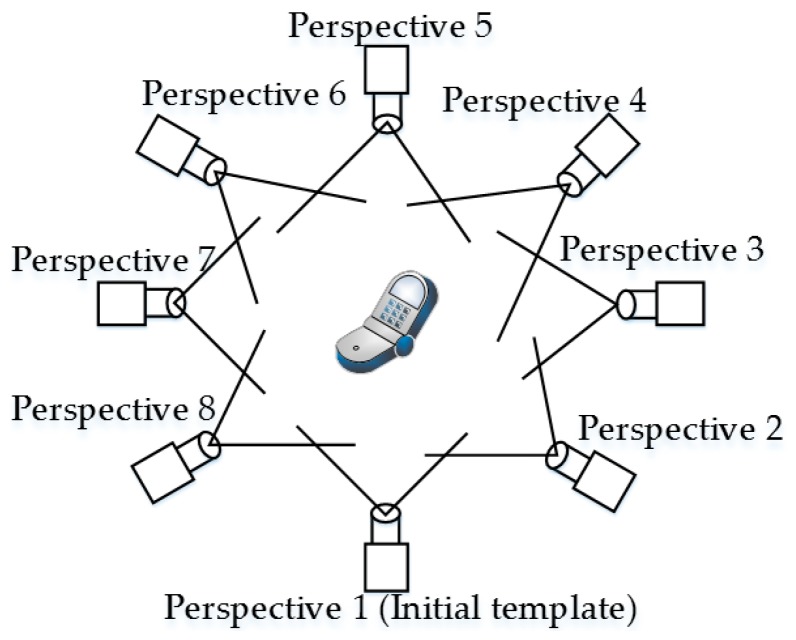
Schematic of multi-view template acquisition

An object template library is first built to fulfill the target and template object matching. The first extracted template is called the initial template, as shown in Figure 8. The object’s point cloud data are equiangularly extracted around its *Z*-axis. The VFH can thus be used to determine the transformation matrix between the initial template and the matched template during the coarse-matching process.

**Figure 9:**
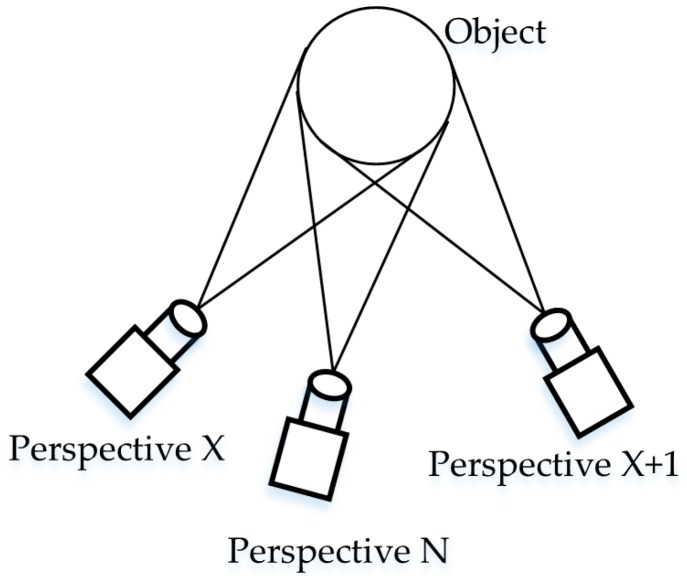
Relationship between various perspectives.

The coarse-matched template object’s pose is selected as the target object’s coarse-matching pose, and the target object should be between two template objects. These two template objects are called templates X and X + 1, respectively, and template X is assumed to be closer to the target template (Figure 9). The coarse transformation matrix ${}_{M}^{X}T$ from template X to the initial template can be determined using a VFH.

### 3.4. Precise-Matching of Target to Template Object

Matrix ${}_{X}^{N}T$ represents the pose transformation matrix from template X to the target object (i.e., perspective N). The precise-matching pose transformation matrix ${}_{M}^{N}T$ can be expressed using ${}_{X}^{N}T$ and ${}_{M}^{X}T$:

(2) $${}_{M}^{N}T={}_{X}^{N}T\cdot {}_{M}^{X}T$$

**Figure 10:**
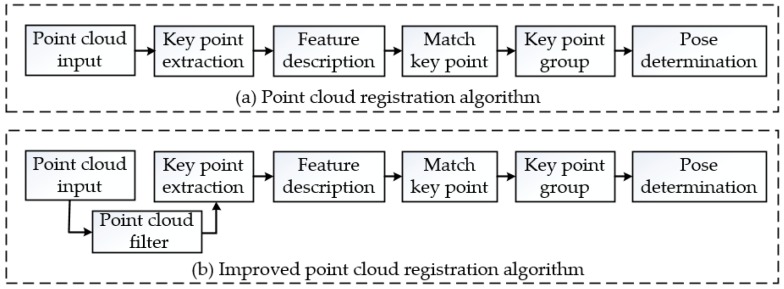
Flowchart of pose determination.

The templates and target objects are rigid, and the volume and shape of their point clouds do not change. Template point cloud set X and target point cloud set N are not equal and do not have an inclusion relationship. Instead, only a few elements are the same in the two sets, which are defined as set C. At least three distinct points should be selected from set C to calculate the transformation matrix ${}_{X}^{N}T$, the process of which is called an improved point cloud registration algorithm, as shown in Figure 10b. Compared with a general registration algorithm, a filter process is added.

A coefficient f (0 < f < 1) is used to select the target point cloud data from C:

(3) $$\left\{ \begin{array}{l} X_{c}=fS_{xy}\left(X\right)\in C\\ N_{c}=fS_{xy}\left(N\right)\in C \end{array}\right.$$

where *X*_c_ represents the filtered point cloud data of the Xth template, *N*_c_ denotes the filtered point cloud data of the Nth object, $S_{xy}\left(X\right)$ represents the projection of the Xth template’s point cloud data in the XY plane, and $S_{xy}\left(N\right)$ represents that of object N’s point cloud data. The coefficient f can be determined using the following steps.

Coefficient λ is defined as the ratio of the intersection area of the Xth and (X + 1)th perspectives to the view angle of the camera α, as shown in Equation (4):

(4) $$\lambda =\frac{\alpha -\frac{2\pi }{k}}{\alpha }$$

where k is the number of templates. The view angle is equal to π for most cases, and Equation (4) can thus be simplified as follows:

(5) $$\lambda =1-\frac{2}{k}$$

In addition, coefficient η denotes that of the Xth and Nth perspectives. Coefficient λ must be less than η, as shown in Figure 7. Because the number of perspectives must be larger than three, coefficient λ is larger than 1/3. The range of f (i.e., λ) can be determined using Equation (6):

(6) $$\frac{1}{3}\leq f\leq \eta$$

A classic key point registration algorithm is used to register *N*_c_ and *X*_c_ to obtain the registration transformation matrix ${}_{X}^{N}T$, as detailed in the following five steps:

(1) Extract the key points from *N*_c_ and *X*_c_ using the SIFT3D algorithm to obtain the key point sets *N*_f_ and *X*_f_ [33];
(2) Calculate the local features using fast point feature histograms (FPFH) of *N*_f_ and *X*_f_;
(3) Group the key points in *N*_f_ and *X*_f_, respectively;
(4) Eliminate incorrect groups using the Hall vote algorithm [34];
(5) Use the sample consensus initial alignment (SAC-IA) algorithm to register *N*_f_ and *X*_f_ and obtain the transformation matrix ${}_{X}^{N}T$.

## 4. Experimental Verification

**Figure 11:**
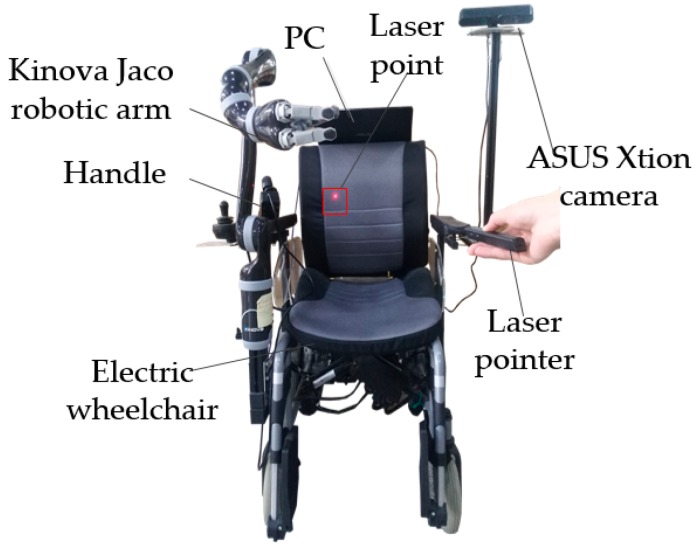
Experimental platform.

The proposed laser point detection and object grasping algorithms were implemented on a WMRA platform, as shown in Figure 11.

### 4.1. Experimental Setup

The platform consists of a 6-DOF Jaco arm produced by Kinova (Montreal, Canada), an electric wheelchair from Vermeiren (Suzhou, China), an RGB-D camera Xtion from ASUS, a handle used to control the Kinova Jaco for demonstration of the grasping capability, an onboard computer, and a laser pointer used for human–robot interaction.

The control system is built based on the robot operation system (ROS) Indigo installed in Ubuntu 14.04. The laser point detection algorithm and the grasping control subsystem are all implemented as ROS nodes on the on-board computer.

**Figure 12:**
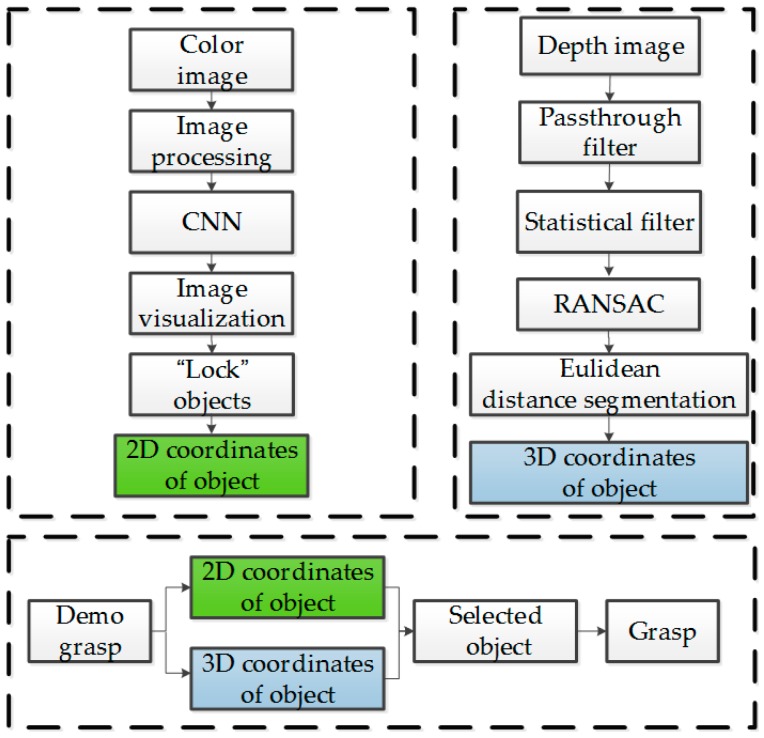
Experimental flowchart.

An experiment flowchart is shown in Figure 12, where the 2D and 3D coordinates of the object being pointed at can be determined using the upper-left and -right sub-flowcharts, respectively. The robotic arm can grasp the object according to the bottom sub-flowchart.

**Figure 13:**
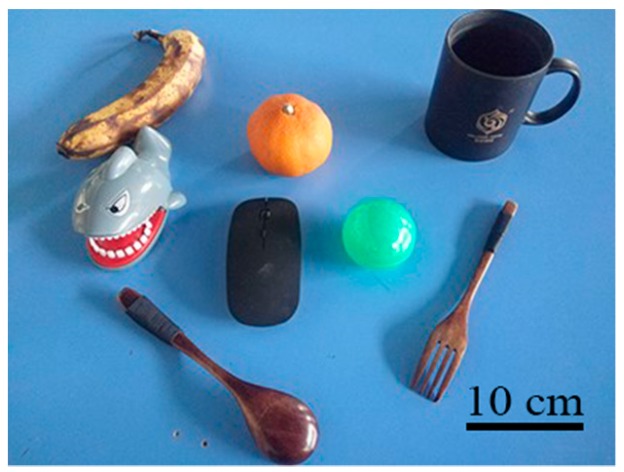
Photograph of experiment objects.

The objects used in the grasping experiments consist of a toy, a banana, a cup, a fork, a ball, a mouse, a spoon, and an orange, as shown in Figure 13. These objects were chosen because they are common household items, and their differences in shape, weight, and color help validate the robot’s performance.

### 4.2. Experiment Results

**Figure 14:**
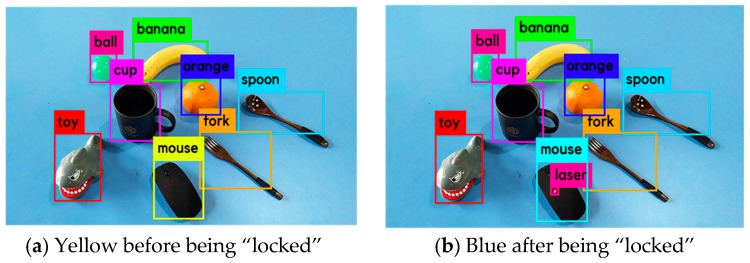
Frame colors of the mouse before and after being “locked”.

An example of a visualization is shown in Figure 14; before the mouse is “locked”, its frame color is yellow (see Figure 14a), after which its frame color changes to blue (see Figure 14b).

A pose estimation experiment was then carried out to evaluate the algorithm. Parameters *E_r_* and *P_r_* were used to evaluate the errors in the estimated rotation matrix and estimated translation matrix, respectively. Here, *E_r_* can be expressed as follows:

(7) $$E_{r}=\frac{\left\|\hat{R}-R\right\|}{\left\|R\right\|}$$

where $\hat{R}$ is the estimated rotation matrix, and *R* is the matrix.

In addition, $P_{r}$ can be given using Equation (8):

(8) $$P_{r}=\sqrt{{\left({\hat{P}}_{X}-P_{X}\right)}^{2}+{\left({\hat{P}}_{Y}-P_{Y}\right)}^{2}+{\left({\hat{P}}_{Z}-P_{Z}\right)}^{2}}$$

where ${\hat{P}}_{X}$, ${\hat{P}}_{Y}$, and ${\hat{P}}_{Z}$ are the estimated X-, Y-, and Z-translation distances, respectively, whereas $P_{X}$, $P_{Y}$, and $P_{Z}$ are the real distances.

Two series of experiments were carried out to thoroughly evaluate the improved pose estimation algorithm, and the evaluation parameters were selected as *E_r_*, *P_r_*, and time consumption *t*. The experiments were carried out 20 times to estimate the poses of the objects using three different algorithms. For each pose, the experiment results were averaged for 20 times, as indicated in Table 1 and Table 2.

Compared with VFH, the VFH + improved key point registration algorithm can estimate the poses of the objects with a higher accuracy at the cost of an increase in the time consumption. When nine templates are used, the calculation error *E_r_* can be reduced from 7.12% to 2.57%, whereas the time consumption t increases from 1.79 to 3.68 s. When six templates are used, the calculation error *E_r_* can be reduced from 21.31% to 3.91%, whereas the time consumption t increases from 2.44 to 4.43 s.

Moreover, if the number of templates is specified, when comparing the VFH + improved key point registration with the VFH + key point registration, the estimation accuracies are almost the same, whereas the time consumption can be reduced significantly (in this case, the decrease in time is approximately 1 s).

**Figure 15:**
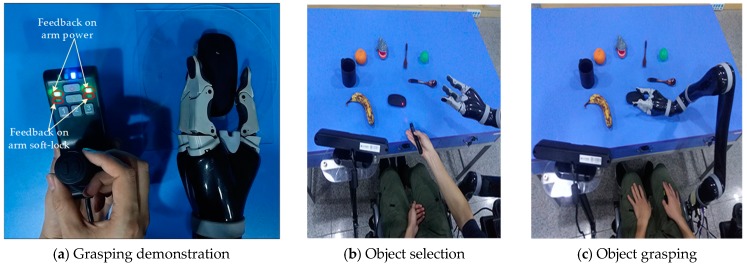
Process of object grasping.

Finally, an experiment on the grasping interaction with a laser pointer was carried out. Figure 15 shows the process of object grasping. The handle was first used to control the arm to grasp the initial template objects manually, and the computer recorded the grasping gesture of the mechanical fingers at the same time, as shown in Figure 15a. The laser pointer was then used to select the object of interest, as shown in Figure 15b. The selected object’s 2D coordinates were estimated using a CNN, whereas its 3D coordinates were determined using the 2D coordinates and depth information from an ASUS Xtion. Given that the grasping pose is already obtained through the algorithm, the robotic arm can grasp the object independently, as shown in Figure 15c, despite the objects being randomly placed during the experiment.

Thirty grasping experiments based on a laser-point interaction were carried out for each object, and the experiment results are as shown in Table 3.

It can be seen from the experiment results that the algorithm successfully realizes the detection of the laser point and achieves an interaction with the robotic arm. In addition, the improved algorithm used in the pose estimation allows the robotic arm to achieve a better grasp. However, the success rate of the grasping was low for the spoon and fork, which was caused by their small geometry. Moreover, the wheelchair being maintained in a stationary position, making the arm unable to reach the object in the template pose during the experiment, is another cause of the low success rate. Because the Kinova Jaco robotic arm is equipped with protection software, the “feedback on arm soft-lock” indicator light on the handle (see Figure 15a) turns red when the object is not properly chosen so as protect the hardware from physical damage.

## 5. Conclusions and Future Work

Based on YOLOv3, this paper presented a laser-point detection method to facilitate assistive grasping with application to a wheelchair-mounted robotic arm. The laser point can be located accurately online, and the object being pointed at can be determined simultaneously through a visualization process. Further, based on both the color and depth information, a precise grasping pose of the robotic arm can be generated using the VFH and the proposed key point registration algorithm. Compared with a commonly accepted method, the time consumption of the pose generation can be reduced from 5.36 to 4.43 s, whereas the pose estimation error is greatly improved from 21.31% to 3.91%.

Structured light was used by the RGB-D camera (Xtion) to get the depth data, while the pattern of light was projected and recorded with a typical CMOS Sensor. Because of the use of light patterns, structured light sensors only produce proper results indoor and environments with controlled light conditions, and the proposed laser pointing scheme performs well in indoor environments.

The selected CNN structure has 106 layers for the detection of many different objects. However, there are many fewer objects that need to be recognized in an indoor environment, and thus a large number of layers is no longer needed. Condensing the convolutional neural network to speed up the object detection method is one of our future areas of focus. Meanwhile, the wheelchair will be controlled to cooperate with the grasping pose determination algorithm to expand the operating space of the robotic arm. 

## Figures and Tables

**Table 1:** Experiment results when six templates are used.

Parameter	True Pose Transformation	Only VFH	VFH + Key Point Registration	VFH + Improved Key Point Registration
*x* (deg)	0	0	2.1356 ± 0.2640	1.6453 ± 0.2135
*y* (deg)	0	0	1.3486 ± 0.1666	−0.8749 ± 0.1135
*z* (deg)	45	60	46.4620 ± 5.7425	47.0037 ± 6.0999
*P_x_* (mm)	10		9.6123 ± 0.5518	10.6575 ± 0.6423
*P_y_* (mm)	10		11.0065 ± 0.6318	9.5700 ± 0.5768
*P*_z_ (mm)	0		0.0034 ± 0.0002	0.3364 ± 0.0203
*t* (s)		2.44	5.36	4.43
*E*_r_		0.2131	0.0414	0.0391
*P*_r_			1.0786	0.8659

**Table 2:** Experiment results when nine templates are used.

Parameter	True Pose Transformation	Only VFH	VFH + Key Point Registration	VFH + Improved Key Point Registration
*x* (deg)	0	0	1.3841 ± 0.1625	−0.3986 ± 0.0491
*y* (deg)	0	0	−0.6849 ± 0.0804	−0.7935 ± 0.0978
*z* (deg)	45	40	45.0064 ± 5.2845	46.5762 ± 5.7422
*P_x_* (mm)	10		10.3794 ± 0.5660	10.8067 ± 0.6188
*P_y_* (mm)	10		9.3428 ± 0.5095	10.3957 ± 0.5952
*P*_z_ (mm)	0		−0.6437 ± 0.0351	−0.5791 ± 0.0332
*t* (s)		1.79	4.66	3.68
*E*_r_		0.0712	0.0220	0.0257
*P*_r_			0.9951	1.069

**Table 3:** Results of grasping experiment.

Grasped Object	Grasping Times	Number of Successes	Laser Point Detection Times
Banana	30	22	30
Orange	30	30	30
Ball	30	30	30
Toy	30	22	30
Mouse	30	24	30
Cup	30	29	30
Fork	30	13	30
Spoon	30	15	30
